# Experiences of rehabilitation services from the perspective of older adults with dual sensory loss – a qualitative study

**DOI:** 10.1080/17482631.2022.2052559

**Published:** 2022-03-29

**Authors:** Elin Lundin, Stephen E. Widén, Moa Wahlqvist, Sarah Granberg, Agneta Anderzén-Carlsson

**Affiliations:** a School of Health Sciences, Örebro University, Örebro, Sweden; b School of Successful Ageing, Örebro University, Örebro, Sweden; c Disability Research, Örebro University, Örebro, Sweden; d The Swedish National Resource Centre for Deafblindness, Lund, Sweden; e Audiological Research Centre, Faculty of Medicine and Health, Örebro University, Örebro, Sweden; f University Health Care Research Centre, Faculty of Medicine and Health, Örebro University, Örebro, Sweden

**Keywords:** Dual sensory loss, healthy ageing, older adults, rehabilitation services, qualitative content analysis

## Abstract

**Purpose:**

To describe the rehabilitation service experiences of older adults with dual sensory loss (DSL).

**Methods:**

Twenty older adults aged ≥65 years with DSL participated in semistructured interviews. Inductive qualitative content analysis was conducted.

**Results:**

The participants’ experiences were classified into three main categories: 1. *Maintaining and regaining function* included experiences regarding interventions compensating for loss of function and medical corrections; 2. *Mastering the situation* described the individuals’ competence of DSL, skills acquisition and taking control; and 3. *Delivery of rehabilitation services* included experiences of encounters with professionals, their attitudes and the organizational impact on accessibility and collaboration.

**Conclusions:**

It was important for participants to regain function and compensate for loss in function and to meet others in group rehabilitation. The professionals’ attitudes were an important factor that affected the participants’ approach to rehabilitation services. Rehabilitation services mainly focused on either VL or HL, not DSL. Based on the participants’ experiences, the rehabilitation services seemed to contribute to the older adults’ well-being, participation in activities and life roles, which is consistent with the WHO’s definition of healthy ageing. The findings can contribute to the development of rehabilitation services for older adults with DSL to meet the diversity of these individuals’ needs.

## Introduction

Combined vision and hearing loss, which is also known as deafblindness or dual sensory loss (DSL; Wittich et al., 2013), is a complex health condition as it affects two of the human senses, vision and hearing (Davidson & Guthrie, 2019; Saunders & Echt, 2007). Vision and hearing are the dominant senses for distance information. In daily life, these two senses perceive information about our surroundings (Kolarik et al., 2016), which is useful in communication, participation, and orientation and mobility (Göransson, 2008). Vision and hearing work synergistically; therefore, when one of these senses is deficient, the other becomes more important. Because both vision and hearing are affected in persons with DSL, it can be difficult to compensate for the loss in one sense with the other (Davidson & Guthrie, 2019; Smith, 2008). The result of DSL is much more complex than the sum of vision and hearing loss (Davidson & Guthrie, 2019; Moller, 2003; Wittich & Simcock, 2019). Therefore, according to both the World Federation of the Deafblind (WFDB, 2018) and the Nordic definition of deafblindness (NVC, 2016), DSL should be considered a distinct health condition. DSL affects persons of all ages and is more frequently associated with syndromic causes in younger populations (Moller, 2003). For older adults (≥65 years), the most common cause of DSL is age-related changes associated with vision and hearing (Saunders & Echt, 2007). According to the global report from WFDB (WFDB, 2018), the prevalence of DSL is 0.2–2% among all ages of the global population. The prevalence of DSL in older adults range between 0.08% (Lundin et al., 2020) and 57% (Heine et al., 2019) depending on the definition of DSL. There is a lack of consensus of how DSL should be defined (Wittich & Simcock, 2019). Some use functional criteria, such as the Nordic definition of deafblindness (NVC, 2016), whereas others use criteria based on objective standardized measurements for vision loss (VL) and hearing loss HL (Schneider et al., 2011). Regardless of the definition used, the prevalence of DSL increases with age (Lundin et al., 2020; WFDB, 2018; Wittich & Simcock, 2019; Wittich et al., 2012).

Health conditions for persons 65 years and older will change during life, and their needs of services will therefore vary (Moody & Sasser, 2018). DSL is one health condition that affects many aspects of older adults’ lives (Guthrie et al., 2016; Heine et al., 2020; Jaiswal et al., 2018). Jaiswal et al. (2018) showed that DSL affected participation in daily life in older persons. Both receiving and conveying information was affected, and older individuals were often dependent on others when walking or travelling outdoors. Difficulties in social interactions could lead to loneliness and isolation (Jaiswal et al., 2018). Participation is essential for an individual’s well-being (WHO, 2015). Therefore, when an older individual’s participation in daily life is affected, it can impact the individual’s potential for healthy ageing. According to the World Health Organization (WHO; WHO, 2015), healthy ageing is “*the process of developing and maintaining the functional ability that enables well-being in older age*.” (WHO, 2015, p. 28). This notion is further explained by three key terms and the interactions among them. The first key term is *functional ability*, which is focusing on having the abilities to be and do things that the individual values. The second key term is *intrinsic capacity*; this notion represents the individual's mental and physical capacities, which can be influenced by the individuals’ health conditions. For example, the participants’ health condition in the context of DSL could influence their ability to walk, see, and hear. The final key term is *environments*, which includes both a narrow and a broader perspective from the individual's home to the society, buildings, relationships, attitudes, health and social policies and the services they offer. Functional ability comprises the interconnection between the intrinsic capacity of the individual and environmental features (WHO, 2015). Further, the key to healthy ageing is “*to live in environments that support and maintain your intrinsic capacity and functional ability*” (WHO, 2019a, p. 1). Rehabilitation for DSL could facilitate healthy ageing.

Rehabilitation services include testing and adaption of assistive devices and functional training with or without devices to maintain or facilitate function for the individual in their daily life activities, life roles and well-being (WHO, 2019b). These rehabilitation services are mainly focused on either VL or HL and not on the complexity and uniqueness of DSL (Möller, 2008; WFDB, 2018). Professionals in rehabilitation services for persons with DSL often possess knowledge on HL or VL but rarely possess knowledge on DSL as a complex and unique health condition (Fraser et al., 2019; WFDB, 2018; Wittich et al., 2012). Therefore, it could be assumed that persons with DSL are at risk of not receiving the rehabilitation services they need. According to WFDB global report (WFDB, 2018), it is important that persons with DSL receive individual rehabilitation services that meet their needs and not services provided exclusively for blind or deaf persons.

Knowledge about rehabilitation services for older adults with DSL is limited. McDonnall et al. (McDonnall et al., 2016) presented results based on a survey to adults 55 years and older living in the USA, including those who had DSL early in life and participants who developed DSL later in life. The focus of the study was to identify adults with DSL, their needs, and challenges and to define priorities for professionals meeting the target group. For those who developed DSL after the age of 21 years, their five most important needs were; “information about devices to improve hearing, transportation, training to use technology, better ability to communicate with healthcare providers and activities to participate in each day” (p. 405). A study from Canada and the USA described rehabilitation of older adults with DSL focusing on the professionals’ experiences. The study showed that beside the professionals’ normal roles according to their occupation, they also reported several other roles that included to help both the individual with DSL and their family members with psychosocial support and to be a navigator through the health care system, etc. (Fraser et al., 2019). A Swedish study (Lundin et al., 2020) based on medical records, described rehabilitation services provided to older adults with DSL from the audiological clinic, low vision clinic (LVC) and eye clinic. The identified rehabilitation services primarily included hearing aids and various magnifiers, and the focus was primarily on either VL or HL. To the best of our knowledge, there is a lack of research on the rehabilitation service experiences of older adults with DSL, specifically focusing on usability in daily life, as well as accessibility. This knowledge is important to develop the DSL specific rehabilitation services to meet the older adults’ needs and preferences. The aim for the present study was to describe the rehabilitation services experiences of older adults with DSL with specific focus on the content, usability in daily life and the environments where the services are offered.

## Materials and methods

### Study design

This study is based on data from a larger project aiming to describe various aspects of severe DSL in older adults (≥65 years old). For the present study, a qualitative explorative design was used (Patton, 2002) focusing on the participants’ own experiences of rehabilitation services. The data were analysed using inductive qualitative content analysis (Elo & Kyngäs, 2008), which is a qualitative descriptive analysis. An inductive approach is well suited to explore fields where previous research is scarce and to generate knowledge of a particular phenomenon (Elo & Kyngäs, 2008).

### Participants

The inclusion criteria were adults aged ≥65 years who were residents in two counties in Sweden and fulfilled the objective standardized measurements for both vision and hearing loss, (i.e., **A**; decimal visual acuity ≤0.3 for distance in the best eye with best correction and a pure tone average [PTA4] for the frequencies 0.5, 1, 2, and 4 kHz, ≥70 dB HL in the better ear, **B**; decimal visual acuity ≤0.5 and [PTA4] ≥70 dB HL, or **C**; decimal visual acuity ≤0.3 and [PTA4] ≥40 dB HL). According to WHO (WHO, 2018), decimal visual acuity ≤0.5 is considered mild VL, and decimal visual acuity ≤0.3 is considered moderate VL. HL is commonly categorized according to the degree of the loss: moderate HL ranges from 41 to 70 dB and severe HL ranges from 71 to 94 dB (Martin et al., 2001). The objective dimensions of VL and HL do not measure how the individuals experience their health condition in daily life. Furthermore, there is no correlation between the degree of VL/HL and how the individual performs in daily life. Individuals with DSL are a heterogeneous group and factors within the bio-psycho-social perspective all play an important role for how the individual functions in daily life (WFDB, 2018).

To be eligible, the persons needed to have experiences of rehabilitation services, be able to master spoken and written Swedish and to express oneself verbally to share experiences and thoughts about the topic. Persons with congenital or a syndrome causing their DSL were excluded because these conditions of DSL begin early in life; therefore, the rehabilitation services provided to these individuals could differ from adults who develop DSL later in life. In February 2019, 78 persons were identified from a previous study population (Lundin et al., 2020) as being eligible and were contacted by mail. Of the 78, 20 persons agreed to participate in the study, 8 declined and 50 gave no response. Of the 20 persons who participated in the study, 11 were women, and 9 were men. Their age ranged from 73 to 96 years (mean = 85), Table I.

**Table I.** Participants included in the interviews (n = 20)

Characteristics		Frequency	
Age (years), median (min—max)		85	(73–96)
Gender, n			
	Women	11	
	Men	9	
Place of residence, n			
	City [≥90,000 inhabitants]	10	
	Town	10	
Living with partner, n		7	
Contact with clinics, n			
	Audiological	20	
	Low Vision	17	
	Vision	20	
Criteria for DSL*			
	A	7	
	B	2	
	C	11	

*Fulfilled the objective standardized measurements for both vision and hearing loss,

**A**, visual acuity ≤0.3 for distance in the best eye with best correction, and a pure tone average [PTA4] for the frequencies 0.5, 1, 2, and 4 kHz, ≥70 dB HL in the better ear.

**B**, visual acuity ≤0.5 for distance in the best eye with best correction, and a pure tone average [PTA4] for the frequencies 0.5, 1, 2, and 4 kHz, ≥70 dB HL in the better ear.

**C**, visual acuity ≤0.3 for distance in the best eye with best correction, and a pure tone average [PTA4] for the frequencies 0.5, 1, 2, and 4 kHz, ≥40 dB HL in the better ear.

### Rehabilitation services in Sweden

According to Swedish legislation, Swedish citizens should have access to rehabilitation services regardless of age, gender, income, and health conditions (The Swedish Health Care Act, 2017). The interventions in rehabilitation services relevant for the present study are typically not provided in specialist centres for people with DSL. Instead they are provided separately for VL and HL. Regarding vision, the rehabilitation is provided by the low vision clinics (LVC) that focus on training skills and techniques for activities in daily life (ADL), such as orientation and mobility, reading and writing, and assistive devices, including different magnifiers, video-magnifiers, refracted glasses and filter glasses. Psychosocial support is also provided by the LVC. In the person’s home, the LVC can provide environmental modifications with extra light and tactile or contrast markups (The National Board of Health and Welfare, 2012). When it comes to HL, persons with DSL can be provided with hearing aids, cochlear implants (CI), psychosocial support, loop systems, alert system to doorbell, telephone and smoke alarm from the audiological clinic. In Sweden, the services and technical devices that are offered to persons with DSL and the costs for them might differ depending on the region where the person lives (The National Board of Health and Welfare, 2012). Rehabilitation services and technical devices are, however mainly subsidized for all citizens in all regions in Sweden.

### Data collection

The first author (EL) previously worked at an LVC and mainly met younger persons with DSL there. She therefore has good experience with meeting with individuals with DSL but limited experience in working with older adults with DSL. The first author collected all the data by individual interviews. Initially, one pilot interview was conducted with a familiar older man who had HL (≥70 dB HL in the better ear) to test the interview guide. No adjustments to the interview guide were made after the interview. The pilot interview was not included in the data set.

The first author contacted all participants before the interview. Information about who the first author was and her background, as well as information about the study and the structure of the interview were shared. All 20 participants decided when and where they wanted the interview to occur. The majority were conducted in the participants’ homes (n = 14), and some interviews were performed at the audiological clinic (n = 6). Before the interview started, the environment was assessed to ensure that the sound and lighting were optimal for the participants. This assessment was performed because the participants exhibit DSL and therefore required environmental adaptation. A loop system was provided if the participants needed it. The aim and the focus of the study were repeated, and the participants were asked if they had any questions about the study before the interview started. The participants were also encouraged to indicate if they wanted a break during the interview. In two of the interviews, a relative attended but did not interfere. One of the interviews lasted 21 minutes, the duration of the other interviews (n = 19) ranged from 44 to 120 minutes (mean 87 min), excluding breaks. Two of the interviews included a coffee break.

A semistructured interview approach was applied with probing questions and follow-up questions, such as “Can you give an example?”, “Can you explain more?”, etc. The framing of the interview guide was inspired by the elaborated person-centred model of audiological rehabilitation (Granberg, 2015) and the Swedish ADL-taxonomy (Törnquist & Sonn, 2017). The interview guide included the participants story about their VL, HL and DSL. The participants were also asked which rehabilitation services were important for them in daily life and why they were important. The guide included themes within rehabilitation services such as psychosocial support, orientation & mobility, communication, information, technical devices, adaptation of the physical environment and ADL (feeding, meal preparation, dressing, clothing care, cleaning, shopping, financial management, social participation). Finally, the participants were encouraged to notify the researcher if they missed any rehabilitation services and if they had tips on areas for improvement.

Additionally, data were collected on participants’ demographic characteristics, including age, gender, place of residence, living with a partner or alone, and which of the three inclusion criteria (A, B or C) for DSL the persons met. Data were also collected regarding which of the rehabilitation clinics (audiological, eye and low vision clinics) the persons received their rehabilitation services from (Table I). The interviews were audio recorded and transcribed verbatim. The first author transcribed the first interview, and research assistants transcribed the other interviews. The transcribed interviews were anonymized during this process, and each interview was assigned a numerical identifier.

### Data analysis

The interviews were imported into the NVivo 12 qualitative data analysis software for Windows (QSR International Pty Ltd. Version 12 Plus). According to Elo and Kyngäs (2008), *open coding, creating categories* and *abstraction* are the three steps used when analysing data.

#### Open coding

To obtain a sense of the data as a whole, each interview was listened to and read through several times by the first author. When reading the transcript, the first author made marginal notes about central topics for the present study. Important quotes discussed by the participants related to their experiences about rehabilitation services were highlighted in the text. Manifest notes for all highlighted quotes were also identified using the data analysis software. The two authors EL and AAC then discussed these notes to ensure that the notes were representative of the data and related to the aim.

#### Creating categories

The notes and the highlighted quotes were read in detail to identify similarities and differences to create categories. Preliminary names for each category related to the content were suggested and discussed between the authors. Using NVivo 12, data were then applied by linking descriptions from the preliminary categories with related content to refined subcategories.

#### Abstraction

The abstraction consisted of organizing the preliminary categories and their respective subcategories to generic categories and finally into meaningful overarching main categories. The name of the subcategories and the higher order categories were related to their content. During the process of abstraction, the authors discussed the content of the categories and reached a consensus of the entire categorization. Finally, direct quotations that were relevant to the aim of the study were identified from the interviews and translated into English. These quotations are presented in the results to give a voice to the participants and for the reader to judge the confirmability of the results.

The first author was responsible for data analysis in close collaboration with the last author (AAC). For the steps of creating categories and abstraction, two co-authors (MW and SW) served as active discussion partners. The research group has an interdisciplinary approach and expertise from different disciplines. All of the members of the group have knowledge about DSL and varied experiences in qualitative methods. Because all the authors had a preunderstanding about the topic, the discussion between the authors had a significant role in analysing and interpreting the data, thus strengthening the trustworthiness. One example from the three steps in the procedure is presented in Table II to allow for assessment of the credibility of the analysis.

**Table II.** An example over the process of abstraction

Unit of analysis →	Node →	Subcategory →	Generic category →	Main category
Interviewer: “and what makes the hearing aid so important to you?” Interviewee: That one can perceive sounds and join in and, well, one functions as a human being. That´s what it´s all about. This is what´s happening when you have one of these [hearing aid]. #3	Hearing aid makes me perceive sounds and allows me to follow [conversations]	Compensating for hearing loss	Compensating for loss in function	Maintaining and regaining function

### Ethical considerations

The study received ethical approval from the Regional Ethics Committee in Uppsala, Sweden (dnr: 2018/379) and was conducted in accordance with the Declaration of Helsinki (World Medical Association, 2018). As older adults with DSL are considered a vulnerable group (Simcock, 2016; Simcock & Wittich, 2019) and the current project covers more than one qualitative research question, exposing the participants only once to an interview was considered to be a good alternative. This accommodation resulted in longer interviews, where pauses were offered. Before the interviews started, a signed informed consent form was obtained from each participant. The participants were informed that they at any time could withdraw their consent without subsequent consequences and that data would be handled with confidentiality.

## Results

The data analysis generated 16 subcategories, seven generic categories and three main-categories. All of the participants’ experiences of rehabilitation services are represented in all main-categories. The main-category M*aintaining and regaining function* included two generic categories and sex subcategories. This main category describes experiences of rehabilitation services focusing on compensating for the older adults’ loss of function of vision and hearing, medical correction of structures in the eye, and the importance of regular check-ups of vision and hearing loss. *Mastering the situation* is the second main-category, which included three generic categories and six subcategories. This main category focuses on experiences of how the participants used their rehabilitation services to gain competence of DSL by acquiring information and increasing knowledge and understanding of their situation. This main category also included the participants’ experiences of acquiring skills and taking control. The last main-category D*elivery of rehabilitation services* included two generic categories and four subcategories. Delivery of rehabilitation services mirrors the participants’ experiences of rehabilitation services focusing on encounters with and attitudes of professionals as well as the organizational impact on rehabilitation, specifically focusing on accessibility and collaboration (Table III). Taken together, the participants’ narratives reveal an image wherein rehabilitation for their DSL is important for maintaining and regaining functions as well as mastering their situation. This perspective was to some degree facilitated or hindered by encounters with and attitudes of professionals as well as the organization of the services. The narratives primarily focused on rehabilitation services for one of the sensory losses and more seldom on services focusing on the complexity of DSL.

**Table III.** Overview of the results

Main-categories	Generic categories	Subcategories
Maintaining and regaining function	Compensating for loss in function	Compensating for vision loss
		Compensating for hearing loss
		Compensating for DSL
	Medical correction for vision loss	Eye drops
		Eye injections
		Eye surgery
Mastering the situation	Competence of DSL	Information and increased knowledge
		Understanding the situation
	Acquiring skills	Meeting with others
		Developing strategies
	Taking control	Taking initiatives
		Taking responsibility
Delivery of rehabilitation services	Impact of professional’s encountering and attitudes	Positive encounters
		Negative encounters
	Organizational impact of importance	Accessibility
		Collaboration

## Maintaining and regaining function

The first generic category, Compensating for loss in function, focuses on the use of different assistive devices and regular check-ups. In the second generic category, Medical correction for vision loss, the participants’ experiences of medical correction for vision loss, such as eye drops, eye injections and eye surgery, are described.

### Compensating for loss in function

All participants had experiences of rehabilitation focusing on compensating for their loss in function of vision and hearing.

Only a few of the participants had experiences of rehabilitation services that focused on interventions in order to compensate for the DSL. They expressed the importance of having tactile alert systems for the doorbell, telephone and smoke alarm. These systems were needed mainly to register sounds when using the hearing aids in combination with a loop system or at night when they participants did not use their hearing aids. The tactile alert system was described as positive because the participants felt safe when they were sleeping or when they used the loop system.

One person discussed her experiences of glasses with attached hearing aids, i.e., “hearing glasses”. She described that the “hearing glasses” were positive for her because the device was “all in one” instead of using both hearing aids and glasses simultaneously. However, she expressed that the disadvantage of the “hearing glasses” was their quality; therefore, she started using separate hearing aids and glasses.

Some of the participants indicated that they were provided different assistive devices in order to compensate their VL by using their hearing instead. For example, some used audio book players because they could not read books anymore. Other used voice recorders to record reminders, and a few used a talking watch.

Among all participants, different magnifiers were the most common devices the persons encountered to facilitate vision. These magnifiers included Closed Circuit Television magnifiers (CCTV), handheld magnifiers and magnifiers with a lamp on a swing arm. Several of the participants described CCTV positively since they could adjust the magnification depending on the purpose of use. In addition, the CCTV provided good contrast and modifiable variation in different colours, and the table of the CCTV made the text or object remain still. However, others expressed that they had difficulties using the table since they had trouble locking the table or that the CCTV was too big and time consuming to use. Others reported that they could not obtain an overview of a whole page because of high magnification.

#14: No, the CCTV works. I´m the one who can´t see. And if I magnify, I only see one letter, it is not possible … And I magnify, magnify. Before, I magnified the whole text, but now if I magnify, magnify a letter and then I can´t …

Interviewer: You only get one letter at time?

#14: Yes. I don´t use [CCTV], before I could, now I lose context and it takes long time. (Man #14)

For those who used handheld magnifiers, they used these magnifiers at home and when out shopping. Several expressed that it was facilitating when the handhold magnifier had a built-in lamp when the surrounding environment was either too light, or too dark. Some participants preferred using magnifier lamp on swing arm to free both hands, but others felt that this device was too large and useless.

The participants described the personal importance of regular check-ups of their vision loss by measuring visual acuity, checking eye pressure and eye screenings. Some of the participants expressed that this practice created a sense of assurance for them because their vision loss was under control. They also described that their glasses were corrected after the check-up.

Some participants used filter glasses for glare sensitivity and to reduce contrast. Filter glasses increased wellbeing because it prevented headaches and improved vision. Some participants used peaked cap or sunglasses instead to reduce glare, and one person expressed that sunglasses reduced irritated eyes. Another rehabilitation service focusing on vision loss was the white cane. Without the cane, participants did not go outside. A few of the participants expressed that they were provided colour marks for the kitchen stove and fitted ceiling lighting. These different environmental adaptations facilitated for the participants when cooking.

All participants had experiences with hearing aids. The majority of the participants emphasized the importance of the hearing aids to facilitate hearing in daily life. Many described that they used hearing aids all day long to perceive sounds, to be able to join in conversations with others, to use the telephone, and to apprehend sound from TV, radio, smoke alarm and doorbell. Hearing aids allowed the persons to participate in ADL and to feel safe in the environment.

“Yes, there are almost no sounds around me unless I use the hearing aids. On the other hand, there cannot be too much surrounding sounds, but still, it must be so I can feel that I am connected with reality. And, it’s the hearing aids that connect me. They are very important to me” (Woman #10)

However, too much noise was not good either. Some of the participants expressed that the hearing aid limited them in their daily life. They described that the sounds changed in a negative way when using hearing aids. Others were worried that the hearing aids could fall out when using a cap to reduce glair or when being physically active. The hearing aid in combination with a loop system was mainly perceived as positive by the participants because the participants could facilitate their hearing and participate in meetings, enjoy the theatre and listen to TV or radio at home. However, some participants expressed that they felt unsafe at home when using hearing aids in combination with a loop system because they were not able to hear other sounds in the environment, such as the smoke alarm, doorbell or someone entering the room.

Some participants described the importance of regular check-ups of their hearing thresholds and that their hearing aids were adjusted accordingly, which improved the ability to receive and interpret sounds. Several of the participants also expressed that they needed help to clean or change battery on the hearing aids because of their VL. Others described that they needed to use glasses instead of handheld magnifiers when they maintained their hearing aids because they needed to use both hands. A few participants described that they visited the audiological clinic for removal of cerumen to be able to continue to use their hearing aids. The participants discussed the importance of these rehabilitation services; otherwise, they would not have been able to facilitate their hearing.

“But, thankfully now when she [the audiologist] did a test [measurement of hearing thresholds], she saw that it [hearing aid] needed to be amplified. So now, when she amplified it, I can hear the voices of my female friends, which were previously difficult to hear”. (Woman #9)

### Medical correction for vision loss

For several of the participants, medical correction was of great importance for them to maintain and regain eye function. The treatments mentioned included eye drops, eye injections and eye surgery. Eye injections for wet macular degeneration were described as having had a positive impact on physical health, and some described the great importance of this treatment. Some participants expressed that their vision stabilized or improved after the medical correction; thus, it was an appreciated treatment. One of the participants described that the eye surgery had been so successful for him that he had regained function in his eye as an eighteen-year-old person. He exemplified that he was now able to see the mark for water on the coffee machine again.

“That coffee machine over there. I thought I would need to return it [to the shop] because I thought there were no grading marks on it. It should had been, so one can see when pouring the coffee. Instead, I have to use a cup to measure. So, I thought that I should go back and return it because I can’t have one of those. When they haven’t noticed that it should be marked. There are such big numbers now on that one. When I got vision. So I’ve discovered a lot now which I haven’t seen. I did not *understand* that my vision was that bad.” (Man #15)

However, for some of the participants, their experiences of eye surgery, such as lens replacement and eye injections, had been a negative experience. They were disappointed that the lens replacement did not improve their vision, and in some cases, their vision worsened. One person described that the eye drops to reduce eye pressure made vision worse.

## Mastering the situation

One experience of rehabilitation services was that it allowed the older person to master the situation better. This was described in three generic categories. The first category, Competence of DSL, focuses on information and increased knowledge about their vision and hearing loss to develop a better understanding of their situation. Through this knowledge and understanding, participants had varying experiences about how they recognized their situation despite uncertainty about their future. The second generic category, Acquiring skills, mirrors the participants’ experiences of meeting others in similar situations and developing strategies from group rehabilitation or via individual services at the LVC. In the last generic category, Taking control, the participants described their individual initiatives and responsibility for their situation. For example, this was achieved by initiating relevant contacts with the clinics or asking for a second opinion.

### Competence of DSL

The participants described that information as well as increased knowledge and understanding of their vision- and hearing loss led to increased competence about their DSL. Several of the participants expressed that they had received information from their ophthalmologist or the optician about their vision diagnoses and the prognosis of their vision loss. Some expressed that they were informed that there was nothing more to do given their severe vision loss. Others described that they had been offered magnifying devices but no glasses or lens replacement because of their vision status. The participants expressed that this information was of great importance for them to be able to recognize their situation, and they were calm about it. However, some reported the opposite. They expressed that this knowledge made them more concerned about their health conditions and the future. One of the participants described that she had difficulties coming to terms with her vision loss, and she had thus been offered to talk to a counsellor, which she had accepted. Some described it difficult to initially accept the use of assistive devices, such as hearing aids and the white cane.

Interviewer: What was it like to get hearing aids?

#11: It was not fun. No, it’s kind of like when I started with the white cane. It was not me. But, I’m very grateful for them being, but this [hearing aids], no.

Interviewer: Doesn’t it feel like you?

#11: No, no. But now, I don’t think about it anymore. But, it was difficult in the beginning.

Interviewer: For how long time was it difficult?

#11: I don’t remember that. It has probably also disappeared a little, I mean, *successively*.

Interviewer: What do you think has disappeared?

#11: This feeling … Actually, I think it has to do with female vanity.” (Woman #11)

### Acquiring skills

The participants described the importance of meeting others and developing strategies for acquiring skills by participating in different course activities focused on their VL or HL. In these meetings, the participants exchanged experiences with others in the same situation, which was much appreciated. The participants also described how they learned strategies and one participant described that a course helped her to master the situation when her vision and hearing loss became more severe.

“That meant tremendously to me. It meant that I went on to live my life. I can´t describe what those courses have meant to me. Because, as I see it now, when my left eye became [worse], how clumsy I became first, although I know how to do it now. After all, I would have never succeeded if I have not received that course. One was given tips on how to avoid cutting oneself, your fingers, one should feel what one did. Yes, it was incredible how valuable it was. Then, he went out, the teacher, for walks [training with white cane with the participants in the course]. It was a week that was *very, very* good. I have attended the course several times. The first time it was free of charge, but I have actually attended twice and paid for the week course myself. Because, I thought I had learned so much. And this with the mark up [for stove etc.], and yes, *everything, everything* one got. You had to go to school, I thought. Yes, yes, I praise that course.” (Woman #18)

However, some participants found that group activities had not helped them. The focus was either not correct or the participant already had the knowledge.

Participants also described that they acquired skills when they were trained to use the cane at the LVC. These acquired skills were very important for the participants; otherwise, they had not dared to go out alone.

### Taking control

The participants expressed how they were mastering their situation by taking control, such as taking initiatives and taking responsibility for their situation. For example, they contacted the rehabilitation services when they felt that their vision or hearing had gotten worse or that they needed support for their devices or new devices. They also asked for a second opinion if they were not satisfied with the results or decisions made by the professionals.

“At first, I was at the low vision clinic and asked, but then he said, no, you don’t have any yellow stain [AMD], and there is no cataract, nothing. And then, I went to this optician over here, where I bought the eyeglasses. He examined me, and I did have cataract, he said. So then, I called the medical center [eye clinic], and then he also told me that I had it [cataract]”. (Woman #17)

Some of the participants expressed that they had been denied eye operations or eye injections and subsequently fought for their cause. Another example of taking control was noted when the participants decided to decline the rehabilitation services they had been offered. One participant expressed that she was feeling too old to receive the rehabilitation services. Another participant described that he had declined CI because of his age and that he was feeling that the society could do something better with the money that would have been spent on the CI.

“But, you know, it would cost over 100,000 [Swedish crowns] just to get it [CI] implanted, and then, the follow up on these [CI]. So, I then considered, by myself, that I´m, after all, I´m 80 years old. And, the question is, how long will I remain. So, I thought it is an unnecessary expense, and better retention of those money, than putting them on my hearing, which is tolerable anyway”. (Man #2)

## Delivery of rehabilitation services

Delivery of rehabilitation is described in two generic categories that focuses on the Impact of encounters with and attitudes of professionals and the Organizational impact of importance for the rehabilitation services. The participants cited positive and negative encounters and attitudes in meetings with professionals. The participants also described their experiences regarding accessibility and collaboration between clinics.

### Impact of professional encounters and attitudes

Several of the participants described that the professionals’ encountering them and their attitudes affected the participants’ continued willingness for rehabilitation services. The participants expressed that it was positive when the professionals took their time in the meeting or when the professionals made it feel quite natural to start using a device.

“Yes, it was, she helped practically, and talked. I don’t know, she was so, yes she, I do not know, she made it seem perfectly natural [laughter]. It was she, who actually helped me overcome it [starting to use …], yes, it was.” (Woman #11)

In some cases, the participants described that they had negative experiences in the meeting with the professionals. For example, they talked about feeling upset when the professionals did not believe in them or when the professionals were ignorant, arrogant or rude. The participants expressed that such behaviours led them to choose another clinic or made them terminate the rehabilitation service. One example of a negative experience was receiving a diagnosis and prognosis by mail instead of receiving information at a personal meeting:

“I got a paper from the eye clinic, what’s his name? [thinking]. I’ve thrown it away now. I saved it for a while because I thought I should. But, then I thought, I throw the shit away. In the letter it was stated that I had the dry [AMD]. I was not called by any physician or anything, only in writing informed that I had the dry. And, that there was nothing to do. As a preventative, I should avoid tobacco, alcohol and eat minerals. That’s all. It hit me hard!” (Man #6).

### Organizational impact of importance

The participants’ experiences of rehabilitation services were affected by the organization. The most often mentioned factors were accessibility and collaboration. Participants expressed that due to their DSL it could be difficult for them to know when it was their turn to see the rehabilitation professionals. For example, it was impossible to see the number on the display or hear their name being called out given the surrounding sounds and their hearing loss.

Furthermore, participants expressed that it was positive when the clinic “called” them on a regular basis instead of when the responsibility was on the participants to contact the clinics. For example, some of the participants could not contact the clinic independently due to the clinic’s use of an answering machine that requested the person to press different numbers to select different services. This task was impossible due to their VL.

The participants also described that it was positive that the audiological clinic or the LVC were available at a short notice when the participants needed to fix their assistive devices or needed to borrow hearing aids when theirs were broken. Without this option, they were isolated and unable to perceive sounds in their environment. Some of the participants expressed that it was useful for them when they could choose which of the clinics in the same county they wanted to visit. They expressed that when they could choose clinic, they were able to be independent; otherwise, they needed a person to follow them given their feelings of a lack of safety in unfamiliar environments.

“And then she wrote a referral, here, up to one [ophthalmologist], yes I was offered to choose, if I should go to the main eye clinic or, if I wanted to go to the smaller eye clinic in my hometown because a physician from the main eye clinic came here as a consultant then. It was most convenient for me to go here locally because I can manage myself without any help. Because, one can locate and see at home [home town], and one can use the travel service then to go to the medical center.” (Woman #18).

The audiological clinic and the LVC occasionally provided house calls, increasing the accessibility of the service. Participants appreciated this service. Furthermore, service was regarded as accessible when the patient could make an appointment independently without any claim for a referral from a physician.

The charges for some of the assistive devices were discussed, and some experienced differences between counties in Sweden. Only one participant paid full cost for her hearing aid because she had chosen to go to a private clinic. She expressed that she was very satisfied with her hearing aid and that the regular audiological clinic could not provide her with a suitable hearing aid. Others described that some of the loop systems were not provided free of charge from the audiological clinic. However, the participants could try them at the clinic or at home before purchase, which was good.

Collaboration between different health care providers, among the three involved clinics, and between clinics in different cities was positively expressed. This cooperation made the participants feel that they were being taken care of. The collaboration was also described on a national level. Some of the participants had positive experiences when their assistive devices suddenly broke when travelling and having them repaired. Another collaboration mentioned was when an ophthalmologist had been in contact with a specialist from another county because the participant had a rare vision diagnosis.

## Discussion

The aim of the study was to describe rehabilitation services from the perspectives of older adults with DSL. The participants provided rich experiences about how they were maintaining and regaining functions through their rehabilitation services. The participants described that these interventions were significant for them to continue with their life-roles and activities in daily life. The participants also expressed the importance of meeting others in a similar situation and developing strategies to master their situation. Further, the participants expressed that the accessibility of rehabilitation services and professional encounters and attitudes impacted their experiences and the outcome of the rehabilitation. This notion was discussed in terms of factors that facilitated or hindered the participants. Despite the DSL, the participants’ focus was mostly on the rehabilitation services for VL or HL.

The study population in this study includes older adults who have a specific health condition, namely DSL, which potentially could affect healthy ageing. Thus, some of the results will be discussed in the light of the key terms of healthy ageing.

Previous literature has noted the rehabilitation services provided to persons with DSL mainly focuses on their VL or HL, not the complexity of DSL or DSL as a unique health condition (c.f Fraser et al., 2019; Möller, 2008; WFDB, 2018; Wittich et al., 2012). This notion was also confirmed in the present study focusing on older adults. It is interesting that the participants did not express any concerns about this notion, although some mentioned feeling more secure when having technical aids supporting their DSL, for example, for perception of environmental activities or sound. They were instead mainly satisfied with their rehabilitation services, except for some accessibility aspects. The WFDB global report (WFDB, 2018) states that if the environments or the assistive devices are not suited for person with DSL, the risk of being dependent of others will increase. These aspects were not identified in this study. It is possible that the experiences of older adults with DSL do not agree with the statement in the global report. The identified discrepancies between the previous literature and the results of this study calls for further research on the benefits and needs of DSL-specific rehabilitation interventions and services.

Several of the participants described the importance of rehabilitation services focusing on compensating for loss in function because these rehabilitative interventions mainly simplified for them in their daily life. Thus, they could continue with meaningful activities despite their health condition of DSL. Therefore, such rehabilitation interventions can be interpreted as related to the key terms of *functional ability* and *intrinsic capacity*, which are both components of healthy ageing (WHO, 2015). According to Mann et al. (2004), using cane, eyeglasses and hearing aids are important to facilitate participation in daily life for older adults in general. In line with this, hearing aids, magnifiers, and white cane, etc., were important tools for the individuals in the present study to compensate for loss in function in DSL, which was also identified in other studies (St-Amour et al., 2019; Wittich et al., 2016). Adapted support and assistive devices are also necessary for persons with DSL to increase participation (Möller, 2008). Assistive devices positively affected the well-being of younger persons with DSL, who felt that they could do things as everyone else when using them. Further, assistive devices could also increase the feeling of security for the individual (Ehn et al., 2019). These finding are consistent with the results of the present study, despite the study population being older adults. According to the WHO (2015), security and participation are essential factors to increase quality of life as people age. Thus, rehabilitation services should focus on maintaining and regaining function. As compensating for loss in function might be beneficial for both functional ability and intrinsic capacity in older people with DSL, it could subsequently affect their course of healthy ageing.

It is interesting that all participants contributed to the results concerning medical corrections as part of the rehabilitation services. According to the WHO (2019b), rehabilitation is integrated with and a complement to medical interventions to create the best conditions that are possible, which was somewhat different from the participants’ view. The medical services and the rehabilitation interventions all had a significant impact on the individual’s health, and most responses were positive. It is possible that this positive impact explains why medical services were mentioned in the interviews when participants were asked about experiences of rehabilitation services. Other possible reasons are that patients might not differentiate between rehabilitation and medical services given that both could affect functions, and they might not be aware of the content of the different concepts and the organization of health care. Another possible explanation is that the participants during the interview recalled their visits to separate clinics and shared their experiences based on this, instead of having the DSL itself in focus.

The result shows that many of the participants had positive experiences from rehabilitation interventions provided in groups with other older adults with similar health conditions. They expressed the importance of meeting with others, with whom they could exchange experiences and develop strategies for mastering their situation and acquiring skills. According to healthy ageing as defined by the WHO (2019a), the importance of meeting others can be interpreted in terms of *functional ability*, which includes the ability of the individual to build and maintain relationships, and meet basic needs, such as “learn, grow and make decisions” (p. 1). Environmental factors can facilitate or limit these abilities of the individual (WHO, 2019a). The positive results regarding group rehabilitation are consistent with a qualitative study of older adults with age-related macular degeneration (AMD) and their experiences with health education programme provided in groups (Eklund & Ivanoff, 2006). Their results showed a feeling of fellowship, benefits of sharing similar challenges and experiences of not being alone. Their group activity included both giving and receiving advice from one another. Health-promoting interventions in groups for independent older adults (≥80) improved the ability of individuals to be independent in ADL three month after the intervention ended, compared to individual interventions. Thus, sharing successful strategies in daily life with others in group activities can be of importance for the individual (Gustafsson et al., 2012) and represents one potential method of rehabilitation for DSL.

In the present study, participants highlighted that encounters with and attitudes of professionals influenced the participants’ further approach to rehabilitation services. According to the concept of healthy ageing included in the key term *environment* (WHO, 2019a), attitudes play a significant role for the individual. Professional encounters and attitudes vary. Clients have expressed that the professionals’ ability to communicate and interact is essential in the meeting and could be regarded as more important than their expertise in the health condition (Taylor, 2020). The participants in the present study did not always explicitly state what they regarded as a good encounter, but they spoke of the result of encounters or emotions awakened by these encounters. According to Taylor et al. (2011), professionals can use different modes when interacting with clients, such as encouraging, collaborating, problem solving, instructing and empathizing modes, which all were implicitly present in the participants’ narratives in this study. Some of these modes are consistent with a study by Fraser et al. (2019) where professionals expressed that they served the role of confident and trusted adviser in meetings with persons with DSL. The results revealed that professionals’ attitude could make it quite natural to start using a device. Consistent with this reasoning, several studies (Johnson et al., 2018; Martin et al., 2005) have showed that clear, effective and trustful communication between the professionals and their clients increase the clients’ compliance with information and strategies that they received in consultations. In comparison, negative encounters and attitudes, such as receiving information about health conditions from the professional by mail and without any further explanations, can increase the risk for persons with DSL of developing depression (Hersh, 2013). Older adults with DSL represent a vulnerable group (Simcock, 2016; Simcock & Wittich, 2019). Therefore, it is important that they receive appropriate rehabilitation services for their complex health condition (Saunders & Echt, 2007; WFDB, 2018) to create good conditions for healthy ageing. However, this does not seem to be sufficient, these individuals also need to be approached with a professional and compassionate attitude. Having the ability to effectively communicate with health professionals is also important for the outcome of consultations (Davidson & Guthrie, 2019).

To gain equal access to health care for older adults, it is important to ensure that environments are accessible to meet their needs (WHO, 2015). For the particular population of this study, it is also important to adapt the environment to the DSL. DSL-friendly environments should include good contrasts, reduced glare, tactile markups, adapted sound and lighting, loop system and to the possibility for personal contact with the person in the clinic reception area (c.f WHO, 2015). The goal of rehabilitation is to promote participation such that the individual is able to continue with life-roles and activities in daily life that the person value (Saunders & Echt, 2012). When the rehabilitation services manage to fulfill these goals, the results indicate that the process of healthy ageing simultaneously seems to increase for older adults with DSL.

The aim of qualitative studies are not to generalize (Polit & Beck, 2012) but to describe the experiences of the phenomenon (Elo & Kyngäs, 2008). According to Bower and Scambler (2007), qualitative research contributes to deeper understanding of phenomenon from the participants’ perspective with a focus on subjective meaning. The applied design facilitated access to unique information from older adults with DSL, a group that has been under-studied. This information can be valuable for policy makers and professionals in developing rehabilitation services for older adults with DSL. Demographic data of the participants as well as information about the Swedish rehabilitation system allow the reader to judge the transferability.

Although DSL likely affecte the ability of individuals with DSL to communicate with others (Davidson & Guthrie, 2019; Jaiswal et al., 2018), 20 persons were willing to participate in interviews. In qualitative studies, the sample size is often discussed to ensure the credibility of the study; it is important to select an appropriate sample size (Graneheim & Lundman, 2004). The saturation of data can indicate the optimal size, and well-saturated data facilitate categorization and abstraction (Guthrie et al., 2004). We believe that we reached data saturation with the available sample who volunteered to participate. During the last interviews, no obvious new information was provided, and the participants are all represented in all the main categories. Although saturation of data was reached, the result may have been different if some of the individuals who were contacted by mail but gave no response participated in the study. These individuals may have had different experiences of rehabilitation services. It is likely that some of these individuals declined to participate due to their DSL health condition or because they had other health conditions in addition to DSL that potentially affected them to a greater extent. Further, in the present study, the analysis process was facilitated by the authors’ interdisciplinary approach and extensive knowledge about DSL.

## Conclusions

It was important for participants to regain function or compensate for loss in function and to meet others in rehabilitation interventions provided in groups. The professionals’ attitudes and encounters importantly influenced the participants’ further approach to rehabilitation services. Rehabilitation services mainly focused on VL or HL, not DSL as complex and unique health condition. Based on older adults’ experiences, rehabilitation services for DSL seem to be consistent with the definition of healthy ageing as stated by the WHO. These rehabilitation services promote older adults’ well-being, participation in ADL and life roles as they age. On a more specific level, the results build knowledge about older adults with DSL and their rehabilitations services. This information can be useful to policymakers and managers developing rehabilitation services to meet the complexity of DSL in this age group.

Although this study has added to the knowledge about rehabilitation services for older people with DSL, more research is needed about the daily life of older adults with DSL. How do they manage? Does DSL affect daily living and life roles? What help do they need to achieve healthy ageing? It is also important to study professionals’ knowledge and experiences about rehabilitation services for older adults with DSL to obtain a more comprehensive picture of rehabilitation services for older adults with DSL. The number of older adults is estimated to increase in the coming decades, both in low and high-income countries. This notion in combination with the increased risk of developing DSL as people age calls for policymakers to engage in rehabilitation services to promote healthy ageing for older adults with DSL. By including older adults in research and highlight their experiences, ageism might be counteracted.

## Data Availability

According to the ethical approval for this study, and for protecting the participants’ confidentiality, data for this study is not available for sharing.
